# Efficacy of dupilumab in the treatment of severe vulvar pruritus associated with lichen sclerosus et atrophicus: a case report

**DOI:** 10.3389/fmed.2024.1422389

**Published:** 2024-06-26

**Authors:** Na Du, Qiuyu Mao, Jingyi Yang, Yiwen Zhang, Xinyan Lyu, Yueyue Li, Wei Min, Jing Xu

**Affiliations:** ^1^Department of Dermatology and Venereology, The First Affiliated Hospital of Soochow University, Suzhou, China; ^2^Minhang Hospital, Fudan University, Shanghai, China; ^3^Department of Dermatology and Venereology, The Affiliated Suzhou Hospital of Nanjing Medical University, Suzhou Municipal Hospital, Gusu School, Nanjing Medical University, Suzhou, Jiangsu, China

**Keywords:** lichen sclerosus et atrophicus, pruritus, biologicals, dupilumab, treatment

## Abstract

Lichen sclerosus et atrophicus (LSA) is a chronic inflammatory skin lesion with an undefined cause. It is more commonly found in the genital area, particularly in adolescents, premenopausal women and postmenopausal women. LSA is difficult to treat and often recurs. The primary treatment for LSA involves the administration of potent topical corticosteroids. Dupilumab is increasingly being used for the treatment of itching in non-atopic dermatitis patients but there are few reports on its use for the treatment of LSA. Here, we present a case of LSA in a 61-year-old woman with extensive vulvar itching. Over four months of dupilumab therapy, significant therapeutic effects were observed, including vulvar skin thinning and pruritus relief without adverse reactions.

## Introduction

Lichen sclerosus et atrophicus (LSA) is a chronic inflammatory skin lesion with an undefined cause, which is believed to be related to autoimmune, genetic factors, infections and other factors. It primarily affects the skin and mucous membranes, and is more commonly found in the genital area, particularly in adolescents, premenopausal women and postmenopausal women, with a higher prevalence in females than males (1, 2). Recent relevant research findings suggest that Th1 cells play a role in the cascade of related inflammatory cytokines (interferon-γ, CXCL9, CXCL10, CCR5, CCL4, CCL5), however, the itching of LSA is difficult to be explained by the cascade of these cytokines (3). The primary treatment for LSA involves the administration of potent topical corticosteroids, with a gradual tapering of the dosage once symptoms have subsided. Additionally, topical calcineurin inhibitors are utilized to mitigate the risk of cutaneous atrophy associated with prolonged use of topical corticosteroids. LSA is difficult to treat and often recurs. Dupilumab is increasingly being used for the treatment of itching in non-atopic dermatitis patients by inhibiting the IL-4/IL-13 signal transduction, but there are few reports on its use for the treatment of LSA. Here, we present a case of LSA in a 61-year-old woman with extensive vulvar itching. Given the known itch-relieving effects of dupilumab (4), we explored its therapeutic potential in this refractory LSA case.

## Case presentation

**Figure 1:**
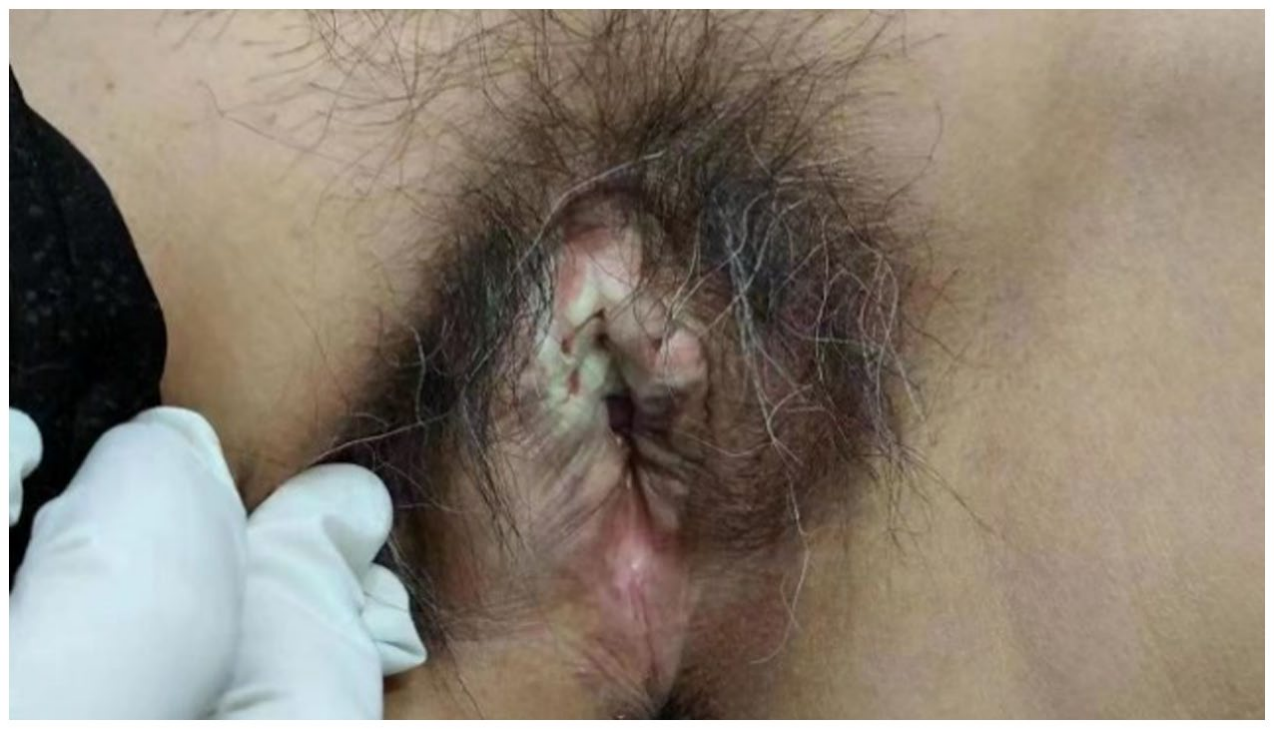
Before treatment, the patient’s vulva was atrophied, sclerotic and white in a large area.

**Figure 2:**
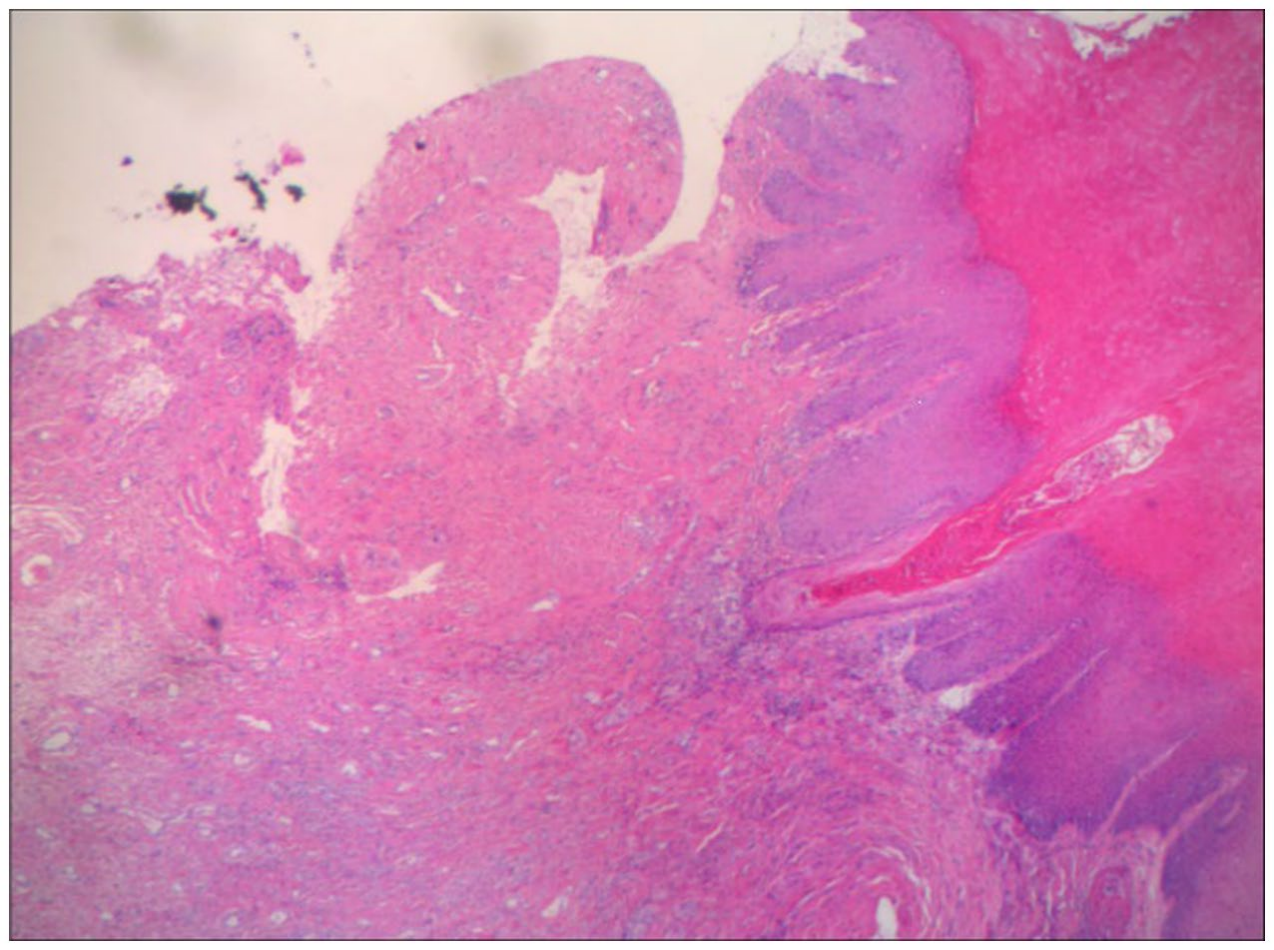
Histopathology of vulvar lesions in VLS patients marked hyperkeratosis, follicular horn plugs, thickening of the epidermal spinous layer, focal liquefaction degeneration of basal cells, and localized homogeneous degeneration of the superficial dermis. Lymphocytes, neutrophils and histiocytes were seen around the blood vessels in the dermis.

A 60-year-old woman came to our dermatology clinic for treatment. One year ago, she experienced severe itching on her right labia majora without any obvious trigger. Two months later, the itching gradually spread to both labia majora and minora. Clinical examination revealed atrophied, white, and hardened mucosa in the labia minora (Figure 1). Topical steroids had no effect on her condition. During the physical examination, we found that the skin of the labia majora and minora had undergone skin atrophy, hypopigmentation, rough thickening, and stiffening. We completed relevant laboratory tests, including anti-nuclear antibody panel、immunofluorescence panel, erythrocyte sedimentation rate, polymyositis panel, but the results showed no obvious abnormalities. There was no special family history. Perform a biopsy on the rash present on her right labia majora showed marked hyperkeratosis, follicular horn plugs, thickening of the epidermal spinous layer, focal vacuolar degeneration of basal cells, and localized homogeneous degeneration of the superficial dermis (Figure 2). Lymphocytes, neutrophils, and histiocytes were observed around dermal blood vessels. These findings supported the diagnosis of LSA.

Initially, the patient was given 0.05% clobetasol propionate cream twice daily for 2 months, combined with oral isotretinoin at a dose of 30 mg per day, once daily. However, there was no improvement in her condition. Subsequently, she received 4 months of oral prednisone at a dose of 30 mg per day and weekly methotrexate at a dose of 10 mg, but her treatment was discontinued due to significant elevation of liver enzymes and steroid-related hypertension. Given significant itch with lichenification, we initiated a therapeutic trial with dupilumab. The patient, weighing 45 kg, received an initial dose of 600 mg of dupilumab, followed by a 300 mg injection every 2 weeks for a total treatment duration of 16 weeks. Throughout this period, the patient continued to apply moisturizer externally, while discontinuing other treatments.

**Figure 3:**
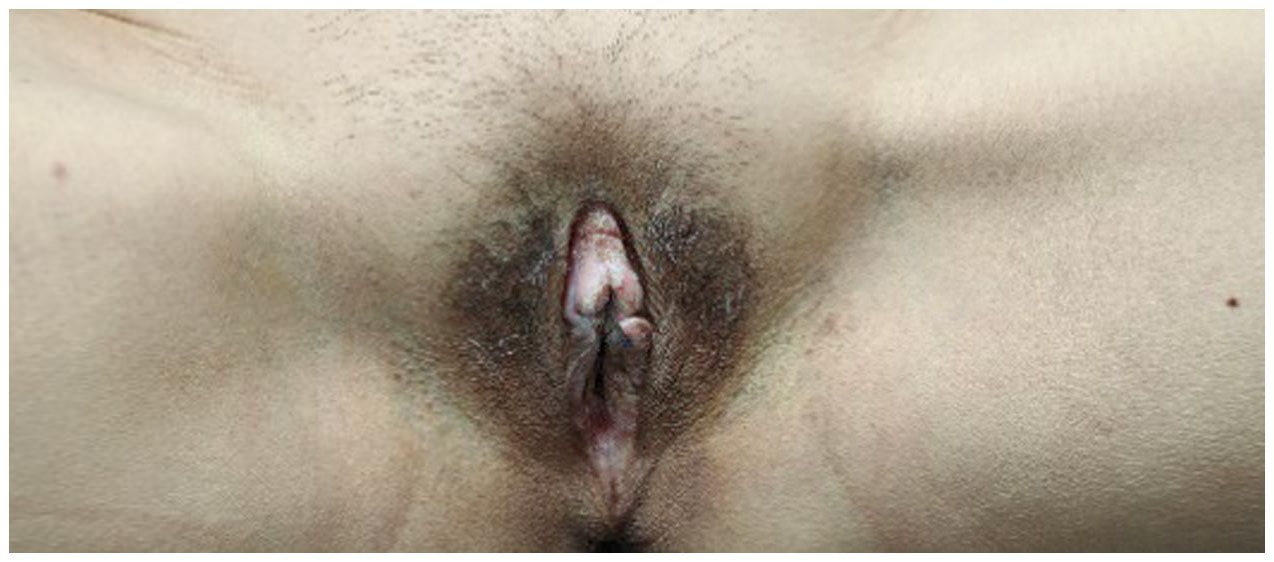
After treatment, the patient’s skin area is significantly reduced and the skin becomes thinner.

Following the initiation of dupilumab treatment, the patient experienced rapid alleviation of pruritus, as evidenced by the peak pruritus numerical rating scale (PP-NRS) decreasing from 9 to 3 within 4 weeks. The quality of life also improved, with a decline in the dermatology life quality index (DLQI) scores from 27 to 15 after 4 weeks of treatment. Over 4 months of dupilumab therapy, significant therapeutic effects were observed, PP-NRS is 1 and DLQI is 3, including vulvar skin thinning and pruritus relief without adverse reactions (Figure 3). Follow-up was 16 weeks till now, the patient exhibits only soy-sized skin lesions on the vulva, with occasional mild itching, managed with local closure. The patients were very satisfied with the treatment results and expressed their gratitude to us. The patient’s treatment process is shown in Table 1.

**Table 1:** Patient treatment regimen.

Previous therapies	Treatment time	Therapeutic effect	PP-NRS	DLQI	Side effect
0.05% clobetasol propionate cream, oral isotretinoin	2 months	No improvement	9	27	The ointment has caused a sensation of burning discomfort in the affected area
Oral prednisone, methotrexate	4 months	Slight improvement	7	25	Significant elevation of liver enzymes and steroid-related hypertension
Dupilumab, moisturizer	4 months	Obvious improvement	1	3	No
Local closure	Till now	Sustained remission	1	2	No

## Discussion

Treatment options for LSA are limited, primarily consisting of glucocorticoid application to affected skin. Other modalities, such as oral glucocorticoids, oral immunosuppressants, dot matrix laser, and focused ultrasound, have shown limited efficacy (4). Given significant itch with lichenification and due to dupilumab’s known efficacy in relieving itch (5), we chose to explore its potential as a treatment option in this LSA case. The remarkable reduction in pruritus and the shrinking size of skin lesions following dupilumab administration suggests its promise as a potential therapy for refractory LSA. The response of vulvar lichen sclerosus to dupilumab may indicate that the IL-4/IL-13 signaling pathway plays a role in the pathogenesis of lichen sclerosus. Although previous studies have shown the presence of Th1 activity in the labia lichen sclerosus, further research is needed to elucidate the potential role of Th2 and IL-4/IL-13 cytokines in vulvar and nonvulvar lichen sclerosus (6). Further research and clinical studies are warranted to corroborate these findings and establish the long-term safety and efficacy of dupilumab in managing LSA.

## Data availability statement

The original contributions presented in the study are included in the article/supplementary material, further inquiries can be directed to the corresponding authors.

## Ethics statement

The studies involving humans were approved by Medical Ethics Committee of the First Affiliated Hospital of Soochow University. The studies were conducted in accordance with the local legislation and institutional requirements. The participants provided their written informed consent to participate in this study. Written informed consent was obtained from the individual (s) for the publication of any potentially identifiable images or data included in this article.

## Author contributions

ND: Investigation, Writing – original draft. QM: Conceptualization, Writing – review & editing. JY: Writing – review & editing, Investigation. YZ: Writing – review & editing, Software. XL: Writing – review & editing, Data curation. YL: Writing – review & editing, Supervision. WM: Supervision, Writing – original draft. XJ: Visualization, Writing – review & editing.
